# Study on the Influence of Low-Price Bid Winning and General Subcontracting Management on the Unsafe Behavior Intention of Construction Workers

**DOI:** 10.3389/fpsyg.2022.822609

**Published:** 2022-04-08

**Authors:** Jinbao Yao, Zhaozhi Wu, Yanan Wen, Zixuan Peng

**Affiliations:** ^1^School of Civil Engineering, Beijing Jiaotong University, Beijing, China; ^2^School of Traffic and Transportation, Beijing Jiaotong University, Beijing, China

**Keywords:** low-price bid winning, general subcontracting management, unsafe behavior intention, mediating effect analysis, multiple regression model

## Abstract

In recent years, there are many reasons for the frequent safety accidents in the construction field. The most controversial and typical one that firmly correlated with China’s national condition is the low-price bid winning and the general subcontracting management, which probably have a great impact on the unsafe behavior intention of workers on the construction site. In order to figure out their internal relation, a quantitative statistical analysis of the unsafe behavior intentions of construction workers in the Beijing area was conducted through the on-site questionnaire considering three main variables, namely, general subcontract management, reasonable low-cost bid winning, and construction experiences. Meanwhile, the correlation, regression, and mediating effects of different influencing factors were analyzed through a regressive model to quantify the impact of each variable on the unsafe behavior intention of construction workers. The results showed that the influence of low-price bid winning on the unsafe behavior intention of on-site workers is faint. This is mainly because, in the case of labor buyer’s market, the actual salary of workers is not relevant to whether the project is awarded at a low price. However, the general subcontracting management has a great impact on the unsafe behavior intention of on-site workers. At the same time, low-price bid winning also indirectly affects the strength of general subcontracting safety management, which has an indirect impact on the unsafe behavior intention of on-site workers. Generally, it is of greater significance to enhance the strength of the general subcontracting management and to formulate relevant regulations to guarantee the safety of construction workers.

## Introduction

The construction industry is one of the most important industries in the world, which provides 10% employment and economic growth (Xu et al., 2018). Because of its dynamic, temporary, and decentralized characteristics (Li et al., 2015), frequent accidents and injuries occur with unsafe behavior as the main factor, and the safety awareness and behavior of construction workers have also become the focus of attention.

Low-price bid winning is a common bid evaluation method, which can save the cost for the tenderee to a certain extent. Therefore, it is widely used in the bidding procurement of county and township government infrastructure construction, the procurement of materials, and the purchase of services. General subcontracting is a unique model in China’s construction industry. It refers to how the owner contracts all of the design or construction tasks to a design unit or a construction unit as the general contractor. The general contractor can subcontract some of its tasks to other contractors to form a structural model of one general design contract or one general construction contract and several subcontracts. However, the low-price bid winning and the general subcontracting management in China are recently considered as the most controversial reasons, accounting for the unsafe behavior intention of construction workers and then leading to the on-site construction accidents. Unsafe behavior intention of construction workers refers to the tendency or possibility of construction workers to take a certain unsafe behavior. It is a psychological process by which construction workers engage in unsafe behavior. China’s Ministry of housing and urban-rural development also issued a document to cancel the bid winning at a low-price because some bidders can win the bid as long as their quotation is low, which leads to more and more construction enterprises pursuing low prices and ignoring quality, laying a great potential safety hazard.

A large amount of research has been conducted in the world in terms of the unsafe behavior intention of construction workers. Earlier studies have shown that human error rather than technical problems have the greatest impact on accidents (Misiurek and Misiurek, 2017). It has been determined that the safety risk tolerance of construction workers is affected by the following three aspects: (1) personal aspect: subjective risk perception (Wang et al., 2016), work experience, skill level (Wang et al., 2016), education level (Meng and Chan, 2020), and self-health incentives (Manjula and De Silva, 2014); (2) organizational aspects: safety management of the project department (Choudhry and Fang, 2008; Wang et al., 2016), training and intervention of front-line leaders (Schwatka et al., 2019), and the project department’s safety guidance (Choudhry and Fang, 2008); (3) external incentives: interaction between colleagues, safety atmosphere of construction projects (Li et al., 2017), and project construction period pressure (Oswald et al., 2013), among others. At the level of personal subjective risk perception, researchers developed the risk perception scale for construction workers (Man et al., 2019) to quantify the impact of workers’ risk perception on their risk-taking behavior (Man et al., 2017, 2019). At the level of organizational aspects, safety education and training for project managers have a significant positive effect on the establishment of workers’ awareness of safety behaviors (Ye et al., 2016; Marín et al., 2019), and the more the project managers involved in front-line safety management, the better the company’s safety environment (Kouabenan et al., 2015; Newaz et al., 2019). As for the external incentives, the interaction among colleagues and the safety atmosphere within the project significantly correlated with the safe behavior intention of construction workers (Yong et al., 2019). Besides, many studies have their own various perspectives concerning the methodology of studying the intention of human behaviors. A machine learning technique, namely random forest modeling, is adopted to scrutinize the non-linear effects of street-scape greenery on the walking propensity of older adults (Yang et al., 2021a). The multi-nomial logit (MNL) model is used to explore the relationship between the rural built environment and the travel mode choice of rural residents (Ao et al., 2020). Global models (linear regression and Box-Cox transformed models) and local models (geographically weighted regression models) are developed to scrutinize the average- (global) and location-specific (local) relationships, respectively, between street greenery and older adults’ walking time (Yang et al., 2021b).

Despite what is mentioned above, there is still a lack of studies concerning the impact imposed by low-price bid winning and general subcontracting management on the unsafe behavior intention of construction workers. Hence, it is significant to figure out their internal relation. In view of China’s national conditions, apart from general subcontracting management and low-price bid winning, this article also added another variable called construction experience to study its impact on workers’ unsafe behavior intentions and to provide the theoretical basis for future safety management on construction sites. The rest of this article was structured as follows. Section “Research Methods” describes the research methods. Section “Results and Analysis” presents the results and analysis. Finally, Section “Conclusion and Expectations” provides conclusions and expectations for future research.

## Research Methods

### 2.1 Background of the Survey

In order to explore the influencing factors of unsafe behavior, this study went deep into the front line of the project and randomly selected five subway projects in Beijing (Anzhenqiao Metro Project Department, Anheqiao Metro Project Department, Anheqiao Second Section Metro Project Department, and Beijing Municipal Subway Project, Tongzhou Metro Project) and one housing construction project (Xierqi Housing Construction). With the cooperation of four graduate students and the person in charge of each project, a face-to-face interpretation of the inquiry records was adopted at the construction site. Multiple surveys with 237 questionnaires were conducted from September 2019 to January 2020. Through sample error elimination, 191 valid questionnaires were screened out.

### 2.2 Design of Questionnaire

The design of the questionnaire is based on the Richter five-point scale, which makes the survey understandable and measurable. The table uses numbers 1–5 as indicators to quantify the respondents’ views on the questionnaire, including total disapproval, disapproval, neutrality, approval, and total approval. The equidistance of indicators can be regarded as sequential continuous variables to facilitate the establishment of mathematical models for later statistical analysis of behavior intentions.

According to relevant literature (Xu et al., 2018), the study has to carry out the following methods:

(1) Complete the preliminary design of the questionnaire, including basic information, analysis of construction workers’ behavior intentions, and analysis of mediation effects.
(2) Discuss and analyze the feasibility and the research ability of the questionnaire with experts who have participated in similar surveys.
(3) Conduct a pre-survey of the questionnaire, conduct a small-scale survey on nearby construction sites, and revise the questionnaire based on the actual situation of the preliminary survey.

The basic information as shown in Table 1 is personal information and descriptive statistics of project experience, including gender, age, education level, years of construction experience, number of similar projects undertaken, and the education and training of this project. In addition to the above factors, through preliminary investigations, different types of workers were considered, such as steel bar, shelf, concrete workers, plumbers, and general workers. The categories of the current projects included subway projects, housing construction projects, municipal projects, and highway projects.

**TABLE 1:** Personal basic information statistics.

Basic information	Option	Number	Proportion (%)
Gender	Man	188	98.4
	Woman	3	1.6
Age	Under 20 years old	6	3.1
	20–29 years old	19	9.9
	30–39 years old	36	18.8
	40–49 years old	71	37.2
	Over 50	59	30.9
Education background	Primary school or below	23	12.0
	Junior middle school	115	60.2
	High school	33	17.3
	Junior college or below	16	8.4
	Bachelor degree or above	4	2.1
Years in construction industry	0–3 years	31	16.2
	4–7 years	48	25.1
	8–10 years	30	15.7
	10–12 years	17	8.9
	More than 12 years	65	34.9
Number of similar projects undertaken	0	22	11.5
	1	23	112.0
	2	45	23.6
	More than 3 similar projects	101	52.9

### 2.3 Questionnaire Assumptions

**FIGURE 1:**
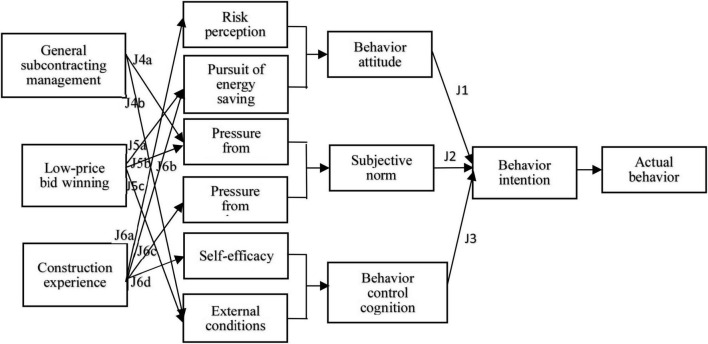
Assumption model.

This study first considered the influence of three dimensions (behavior attitude, subjective norm, and behavior control cognition) composed of six factors (risk perception, pursuit of energy-saving, pressure from managers, pressure from workmates, self-efficacy, and external conditions) on behavior intention (shown in Figure 1). Three assumptions were made according to the reference (Xu et al., 2018) for the branches J1, J2, and J3 as mentioned in Table 2.

**TABLE 2:** Questionnaire assumptions.

Item	Assumption
J1	Individual pursuit of energy saving causes unsafe behavior intention, while individual risk perception promotes safe behavior intention
J2	Pressure from managers and workmates promotes safe behavior intention
J3	External conditions and workers’ self-efficacy promote safe behavior intention
J4	General subcontracting management indirectly affects unsafe behavior intention through the pressure from managers (J4a) and external conditions as mediating variables (J4b)
J5	Low-price bid winning takes the pursuit of energy saving (J5a), pressure from managers (J5b), and external conditions (J5c) as mediating variables and has an indirect impact on unsafe behavior intentions
J6	Construction experience takes pressure from workmates (J6a), risk perception (J6c), pursuit of energy saving (J6b), and self-efficacy (J6d) as mediating variables to have an indirect effect on unsafe behavior intentions

The analysis of the mediating effect was the second part of the hypothetical content of this article. According to China’s national conditions, three variables, including low-price bid winning, general subcontracting system, and the construction experience of workers, had been added to the assumption model (branches J4, J5, and J6 shown in Table 2 and Figure 1). The mediating effects from three variables, through six mediating variables, to the behavior intention were analyzed. Table 3 shows the questionnaire items set for the assumption model.

**TABLE 3:** Questionnaire items.

Items	Questions
Risk perception	Are you familiar with the on-site risks that may be harmful to your safety?
	Do you agree that wearing the protective articles can effectively avoid accidents?
Pursuit of energy saving	Do you agree that safety protective articles are uncomfortable to wear?
	Do you agree that it will be tiring to wear the protective articles for operation?
Pressure from managers	Will the manager correct you for not wearing the protective articles?
	Will the manager punish you for not wearing the protective articles?
Pressure from workmates	Do you obey to wear the protective articles when working with your workmates?
	Do your workmates remind you of wearing the protective articles?
Self-efficacy	Do you think you can wear protective articles correctly?
	Do you find it difficult to work when wearing protective articles?
External conditions	Are adequate protective articles provided for you?
	Are you satisfied with the quality of the protective articles?
Behavior intention	Will you not wear protective articles in the next two weeks because it is bulky?
	Do you plan to wear protective articles all the time in the next two weeks?
	In the next two weeks, will you wear protective articles according to the expectations of workmates and the requirements of managers?
	In the next two weeks, will you not wear protective articles to save time?
Construction experience	Have you been injured caused by not wearing protective articles?
	Have you been punished for not wearing protective articles?
	Have you been wearing protective articles correctly in the past 2 months?
	Have you met with safety problems caused by not wearing protective articles?
Low-price bid winning	For more earning, will you save time and cost without considering your own safety?
	Despite the low project price, the manager will correct and punish the behavior of not wearing protective articles during construction?
	Despite the low project price, are adequate protective articles provided for you?
	Despite the low project price, are the quality of protective articles satisfactory for you?
General subcontracting management	Is general subcontracting management effective for the supervision of not wearing the protective articles?
	Does general subcontracting management focus on the punishment of the unsafe behavior?
	Are adequate protective articles provided for you by the general subcontracting company?
	Are you satisfied with the quality of the protective articles provided by the general subcontracting company?

### 2.4 Analysis Methods and Basis

#### 2.4.1 Exploratory Factor Analysis

Exploratory factor analysis (EFA) is a technique used to find out the essential structure of multivariate observation variables and to process and reduce the dimensions. EFA can synthesize the complicated topics into a few core factors. Using the idea of dimension reduction, starting from the study of the internal dependency of the correlation matrix of the original variables, some variables with complicated relations are expressed as a linear combination of a few common factors and special factors that only have an effect on a certain variable. Namely, the information of original variables is recombined in search of the common factors affecting the variables for the simplification of their intern relations.

#### 2.4.2 Multiple Regression Analysis

Regression analysis is the use of linear relationship model interpretation and prediction. For example, this study assumed six factors (risk perception *X*_1_, pursuit of energy-saving *X*_2_, pressure from managers *X*_3_, pressure from workmates *X*_4_, self-efficacy *X*_5_, and external conditions *X*_6_) as the cause of unsafe behavior y, then the multiple regression equation of dependent variable is as follows:


$$\text{Y}=b_{1}\,X_{1}+b_{2}\,X_{2}+b_{3}\,X_{3}+b_{4}\,X_{4}+b_{5}\,X_{5}+b_{6}\,X_{6}+\alpha$$

where *a* is a constant coefficient.

Through regression analysis, we determined the explanatory degree of each variable to the dependent variable in the regression model, established a regression equation, and checked whether the proposed explanatory variable can be used to explain the dependent variable.

#### 2.4.3 Bootstrap Mediating Effect Analysis

Bootstrap is a non-parametric resampling program, which simulates *n* virtual investigations by resampling the existing samples for *n* times and makes a mediating effect analysis on each simulated data to obtain *n* mediating effect values. The *n* mediating effect values were sorted, and the values less than 2.5% and greater than 97.5% were removed to obtain a 95% CI. If the CI does not include 0, it means that, if the results were significant, mediating effect exists.

## Results and Analysis

### Results of Reliability Tests

For statistical analyses, SPSS 25 software was used to test the reliability of 16 questions (12 questions quantifying six factors, namely risk perception, pursuit of energy-saving, pressure from managers, pressure from workmates, self-efficacy, and external conditions, while four questions quantifying behavior intention) and transpose the negative question.

Cronbach’s *a* and the number of items are 0.683 and 16, respectively. In general, Cronbach’s *a* value <0.6 is considered as unsatisfactory, while 0.6–0.7 is considered as acceptable reliability. Therefore, the data of this study has passed the reliability test, which indicated that there is inherent consistency among all items of the scale and that it can be further analyzed.

### Results of Validity Test and Factor Analysis

#### 3.2.1 Validity Test

Validity analysis measures whether the comprehensive evaluation system can accurately reflect the purpose and requirements of the evaluation. The higher the validity, the more the measurement result can show the feature to be measured. Additionally, the factor analysis can only be started after passing the KMO and the Bartlett sphere test in the validity analysis. The validity of the questions in this questionnaire is analyzed.

The KMO and the Bartlett sphere test values are shown in Table 4. The KMO value above 0.7 is suitable for factor analysis. The significance probability <0.05 of the Bartlett spherical test statistical value can indicate that a correlation exists between each variable. The significance of this questionnaire is 0, which has passed the significance test and is suitable for factor analysis.

**TABLE 4:** KMO and Bartlett tests.

KMO and Bartlett spherical test	Behavior perception intention
Measure of sampling adequacy of KMO	0.721
**Bartlett spherical test**	
Approximate chi-square	357.598
Degree of freedom	66
Significance	0.000

#### 3.2.2 Factor Analysis

Table 5 is the factor component matrix after rotation, from which six factors related to behavior can be obtained, and the six common factors are risk perception, the pursuit of energy-saving, pressure from managers, pressure from workmates, self-efficacy, and external conditions.

**TABLE 5:** Factor component matrix after rotation.

	External conditions	Pursuit of energy saving	Risk perception	Pressure from workmates	Self efficacy	Pressure from managers
The project has a strong safety atmosphere	0.522					
The project department provides sufficient protective articles	0.807					
Project protective articles meets safety regulations	0.809					
Uncomfortable when wearing protective articles		0.892				
Wearing protective articles is tiring		0.886				
Whether workers familiar with construction risks			0.846			
Wearing protective articles can avoid accidents			0.710			
Keep consistent with the surrounding workers who do not wear protective articles				0.605		
When working with workmates, they should abide by the rules of wearing protective articles				0.893		
Even if it is difficult to wear protective articles, it is guaranteed to wear protective articles					0.407	
It is not difficult to wear protective articles					0.932	
The foreman supervises and corrects the behavior without protective articles						0.939
Variance contribution rate	15.146%	13.512%	11.872%	11.446%	9.559%	9.424%
Cumulative variance contribution rate	15.146%	28.658%	40.530%	51.979%	61.535%	70.959%

*Extraction method: Principal component analysis.*

*Rotation method: Caesar normalized maximum variance method.*

*The rotation has converged after five iterations.*

Through factor analysis of this questionnaire, each behavior question in the questionnaire is regarded as an independent variable. The extraction method is the principal component analysis, and the rotation method is Caesar’s normalized maximum variance method, which converges after five rotations. Meanwhile, independent variables with low contribution rates were eliminated.

The final revised questionnaire is concentrated into six factors, which is consistent with the above six factors. Among them, six factors can explain 70.959% of the information of the questionnaire, the variance interpretation effect was good, the information loss of exploratory factor analysis was small, and the model fitting was better. It means that the design questionnaire can fully cover the essence of six factors and is reasonable for the analysis.

### 3.3 Correlation Analysis

Through the correlation analysis, we can determine the correlation between two factors in each dimension and then determine the fit between each dimension and each factor. The correlation of each dimension is shown in Table 6.

**TABLE 6:** Dimension correlation.

		Pursuit of energy saving	Pressure from workmates	External conditions
Risk perception	Pearson correlation	0.021		
	Significance	0.769		
	Number of cases	191		
Pressure from managers	Pearson correlation		0.371**	
	Significance		0.000	
	Number of cases		191	
Self-efficacy	Pearson correlation			0.235**
	Significance			0.001
	Number of cases			191

**At 0.01 level (two tail), the correlation was significant.

(1) The significance of the risk perception and the pursuit of energy-saving in behavior attitude dimension is 0.769 > 0.005, which does not meet the significance hypothesis, and there is no obvious correlation between them.
(2) The significance of pressure from managers and workmates in the subjective norms dimension is 0, and the correlation is significant at 0.01 level. It means that the pressure will move from the managers to the workmates and then influence the behavior intention.
(3) The significance of self-efficacy and external conditions in the behavior control cognition dimension is 0, and the correlation is significant at 0.01 level. It means that the external conditions will interact with the self-efficacy in reality. For example, whether the protective articles are provided and of high quality or not will determine whether the workers have faith in conducting safely in the construction sites.

### *3.4* Regression and Mediating Effect Analysis

#### 3.4.1 Regression Analysis Between Common Factors and Behavioral Intention

In the questionnaire, the multiple regression analysis is used to test the explanatory degree of each variable (six common factors) to the dependent variable (behavioral intention).

(1) Table 7 shows that the regression significance of the six independent variables to behavior intention is less than 0.05, which was significant. It means that the six independent variables are strongly linear with the behavior intention.

**TABLE 7:** Regression analysis.

	Behavior intention	Risk perception	Pursuit of energy saving	Pressure from managers	Pressure from workmates	Self- efficacy	External condition
Behavior intention		0.001	0.044	0.007	0.000	0.011	0.001
Risk perception			0.384	0.000	0.003	0.016	0.022
Pursuit of energy saving				0.144	0.048	0.080	0.053
Pressure from managers					0.000	0.000	0.000
Pressure from workmates						0.000	0.000
Self-efficacy							0.000
External conditions

(2) Some independent variables were further post-checked. The coefficient estimation results (Table 8) point out that the pressure from workmates, risk perception, and external conditions have strong explanatory power for behavior intentions (Beta values are 0.176, 0.161, and 0.145, respectively). It means that these factors have a positive impact on the behavior intention of and lead to the safe behavior of construction workers on site.

**TABLE 8:** Collinearity and model coefficients.

	Unstandardized coefficient	Normalization coefficient	*t*	Significance	Collinearity statistics	
	B	Beta			Allowance	VIF
(Constant)	1.845		3.561	0.000		
Risk perception	0.210	0.161	2.262	0.025	0.911	1.097
Pursuit of energy saving	−0.146	−0.173	−2.510	0.013	0.974	1.026
Pressure from managers	0.001	0.001	0.013	0.990	0.715	1.398
Pressure from workmates	0.183	0.176	2.324	0.021	0.811	1.233
Self-efficacy	0.093	0.078	1.050	0.295	0.848	1.180
External conditions	0.138	0.145	1.933	0.055	0.824	1.213

(3) The Beta coefficient for the pursuit of energy-saving is negative, which means that the intention of workers to not wear protective articles in the future is more obvious for the purpose of energy saving. This is consistent with the assumption in Section “Questionnaire Assumption.” It means that the pursuit of energy-saving has a negative impact on the behavior intention and leads to the unsafe behavior of construction workers on site.

In order to avoid the influence of a strong correlation between common factors on regression results, the co-variates among explanatory variables are considered, and the collinearity of the detection model is shown in Table 8. Among them, the *T*-test results show that the Beta values for the pressure from managers and self-efficacy are low, and the *P*-values are both greater than 0.05, which is statistically insignificant. One of the main reasons is that the variance inflation factor (VIF) value for the pressure from managers and self-efficacy in the regression model is relatively large, and there is a certain correlation between independent variables. Therefore, the significance of the pressure from managers and self-efficacy is not obvious, and there will be some errors. However, the explanatory variables that do not reach the significant level cannot be ignored, and all variables need to be concerned. The final regression model can be shown in the following formula:


$$\text{Y}=1.845+0.21\,X_{1}-0.146\,X_{2}+0.001\,X_{3}+0.183\,X_{4}+0.093\,X_{5}+0.138\,X_{6},$$

where Y is behavior intention, *X*_1_: risk perception, *X*_2_: pursuit of energy saving, *X*_3_: pressure from managers, *X*_4_: pressure from workmates, *X*_5_: self-efficacy, and *X*_6_: external conditions.

Generally speaking, the VIF of all explanatory variables in the questionnaire is less than 2, and the overall multi-collinearity phenomenon is not obvious. Further overall test results of the model (Table 9) indicate that the regression effect reaches a significant level (*F* = 5.242, *p* = 0), which is of statistical significance.

**TABLE 9:** ANOVA variance analysis.

	Sum of squares	Degree of freedom	Mean square	*F*	Significance
Regression	21.673	6	3.612	5.242	0.000
Residual	126.803	184	0.689		
Total	148.476	190			

*Dependent variable: behavior intention.*

*Predicted variables: risk perception, pursuit of energy-saving, pressure from managers, pressure from workers, self-efficacy, and external conditions.*

#### 3.4.2 Analysis of Mediating Function

**FIGURE 2:**
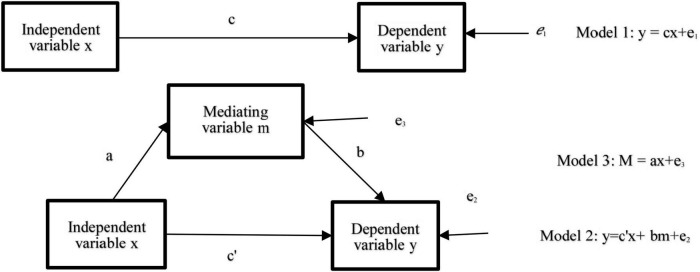
Schematic diagram of mediating effect.

In order to explore the mediating variable M, through which X influences Y, the mediating effect test is carried out. The relationship diagram is shown in Figure 2. The mediating effect is divided into the following three models:

Model 1: The regression analysis of an independent variable x and a dependent variable y to obtain the total effect c value.
Model 2: The regression analysis of an independent variable x, a mediating variable m, and a dependent variable y to obtain direct effect value c’ and mediating effect value b.
Model 3: The regression analysis of an independent variable X and an intermediate variable M to obtain mediating effect value a.

Among them, the regression coefficient a × b of the above model is an indirect effect; if it is significant, it means that the mediating effect exists. In this study, the Bootstrap sampling method is used to test whether the 95% CI of the regression coefficient a × b contains the number 0. If it does not contain the number 0, it has a mediating effect, otherwise, it does not have a mediating effect. Because the number of samples in this questionnaire is less than 500, Bootstrap sampling is carried out 5,000 times to analyze the mediating effect, and the analysis results are shown in Table 10.

**TABLE 10:** Analysis of the mediating effect.

Terms	*c*	*a*	*b*	a*b Estimate	a*b	c’	Test conclusion	Effect proportion
	Total effect			Mediating effect	(95%BootCI)	Direct effect		
General subcontracting management- Pressure from manager-Behavior intention	0.094	0.132*	0.130*	0.017	0.006–0.057	0.077	Full mediation	100%
General subcontracting management-External conditions-Behavior intention	0.117	0.152*	0.193**	0.029	0.000–0.076	0.087	Full mediation	100%
Low-price bid winning-Pursuit of energy saving- Behavior intention	0.362**	−0.015	−0.101	0.002	−0.019 to 0.024	0.360**	Insignificant mediation	0%
Low-price bid winning-Pressure from manager- Behavior intention	0.296**	0.270**	0.047	0.013	−0.033 to 0.057	0.283**	Insignificant mediation	0%
Low-price bid winning-External conditions Behavior intention	−0.126*	−0.186**	0.173**	−0.032	−0.097 to −0.009	−0.094	Full mediation	100%
Construction experience-Risk perception-Behavior intention	0.251**	0.079	0.257**	0.020	−0.005 to 0.062	0.230**	Insignificant mediation	0%
Construction experience-Pursuit of energy saving-Behavior intention	−0.316**	−0.222*	−0.154**	0.034	0.006–0.071	−0.350**	Suppression effect	0%
Construction experience-Self efficacy-Behavior intention	0.251**	0.088	0.161	0.014	−0.006 to 0.052	0.236**	Insignificant mediation	0%
Construction experience-Pressure from workmates-Behavior intention	0.251**	0.133*	0.219**	0.029	0.000–0.075	0.221**	Partial mediation	11.663%
General subcontracting management-Risk perception-Behavior intention	0.091	0.121*	0.283**	0.034	−0.003 to 0.083	0.056	Full mediation	100%
Low-price winning bid-Pressure from workmates-Behavior intention	0.447**	0.335**	0.146*	0.049	0.002–0.096	0.398**	Partial mediation	10.932%
Construction experience-Pressure from managers-Behavior intention	0.251**	0.243**	0.100	0.024	0.005–0.068	0.226**	Partial mediation	9.717%

*p < 0.05; **p <0.01.

**FIGURE 3:**
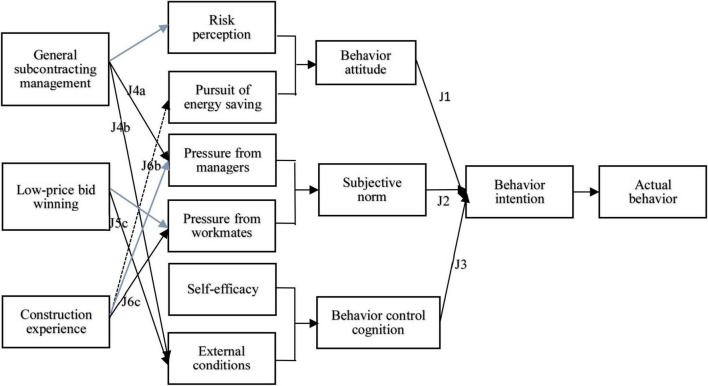
Modified model diagram.

(1) General subcontracting management has an indirect effect on unsafe behavior intentions by influencing workers’ risk perception, pressure from the manager, and external conditions as mediating variables. The newly added risk perception as mediating variable indirectly affects workers’ unsafe behavior because the pressure exerted by the general subcontracting manager on workers accounts for a slight difference in workers’ risk perception level on the spot.
(2) Low-price winning bid influences the unsafe behavior intention of construction workers with the external conditions as mediating variables, because low-price winning bid will, during the general subcontracting management, reduce the cost of safety protection articles, raising the potential impact of workers not wearing safety protection articles.
(3) Construction experience influences unsafe behavior intention of construction workers with the pursuit of energy-saving, pressure from managers and workmates as mediating variables, among which construction experience has a certain suppression effect on behavior intention for the pursuit of energy-saving. This is slightly different from the assumption in Section “Questionnaire Assumption,” so the map is modified (as shown in Figure 3). The gray line is the new regression relationship and the mediating effect in the process of mediating analysis.

(4) There are more mediating factors for the general subcontracting management than the low-price bid winning to influence the behavior intention. Hence, general subcontracting management has a greater impact on the behavior intention. Besides, external conditions serve as the mediating factors at the same time for general subcontracting management and low-price bid winning, so low-price bid winning can, to some extent, influence the strength of general subcontracting management to account for the unsafe behavior attention, for example, by reducing the supply and neglecting the quality of protective articles (external condition) owing to the low project price. Despite the lack of money because of the low-price bid winning, the strength of general subcontracting management still can still be enhanced through other mediating variables, for example, by reinforcing the unsafe behavior supervision and punishment (pressure from managers) and by raising the workers’ awareness of safety (risk perception).

## Conclusion and Expectations

### Conclusion

(1) The research verifies the assumed behavior intentional model based on the mediating variables concerning behavior attitudes, subjective norms, and behavior control cognition. On this basis, the model is workable for the analysis of the unsafe behavior intention by adding the factors including low-price bid winning and general subcontracting management.
(2) The influence of low-price bid winning on the unsafe behavior intention of on-site workers is faint, mainly because, in the case of labor buyer’s market, the actual salary of workers is not relevant to whether the project is awarded at a low price.
(3) General subcontracting management has a greater impact on the unsafe behavior intention of construction workers through the mediating factors including pressure from managers, external conditions, and individual risk perception. At the same time, low-price bid winning also indirectly affects the strength of general subcontracting safety management, which has an indirect impact on the unsafe behavior intention of on-site workers.
(4) Compared to the low-price bid winning, reasonable general subcontracting management is of greater significance. At the national level, strict supervision and punishment measures are supposed to be adopted for the general subcontracting management to reduce the unsafe behavior of construction workers.

### Expectations

The survey only focuses on the projects under construction in Beijing, which has certain limitations, mainly in the following aspects:

(1) There are some differences in the supervision of projects in different regions. The overall cultural quality of workers and the treatment of safety atmosphere in the project department have a certain impact on the measurement of the questionnaire survey.
(2) Most of the questionnaires are about subway projects, while the research on housing construction projects and highway projects is relatively lacking. The follow-up research will cover more types of projects.
(3) No major safety accident has occurred in the surveyed project in this questionnaire, and this questionnaire is intended to measure workers’ unsafe behavior and unsafe behavior intention. Since no unsafe accident has occurred, workers’ response only depends on their inner thoughts rather than personal experience. Therefore, the follow-up research will focus on the projects that have occurred on safety accidents or unsafe behaviors.

## Data Availability Statement

The raw data supporting the conclusions of this article will be made available by the authors, without undue reservation.

## Ethics Statement

Ethical review and approval was not required for the study on human participants in accordance with the local legislation and institutional requirements. Written informed consent from the patients/ participants was not required to participate in this study in accordance with the national legislation and the institutional requirements.

## Author Contributions

JY: conceptualization, methodology, and writing – original draft. ZW: methodology, programming, and writing-improvement. YW and ZP: investigation and data collection. All authors contributed to the article and approved the submitted version.

## Conflict of Interest

The authors declare that the research was conducted in the absence of any commercial or financial relationships that could be construed as a potential conflict of interest.

## Publisher’s Note

All claims expressed in this article are solely those of the authors and do not necessarily represent those of their affiliated organizations, or those of the publisher, the editors and the reviewers. Any product that may be evaluated in this article, or claim that may be made by its manufacturer, is not guaranteed or endorsed by the publisher.
